# Lack of efficacy of blueberry in nutritional prevention of azoxymethane-initiated cancers of rat small intestine and colon

**DOI:** 10.1186/1471-230X-9-67

**Published:** 2009-09-16

**Authors:** Frank A Simmen, Julie A Frank, Xianli Wu, Rijin Xiao, Leah J Hennings, Ronald L Prior

**Affiliations:** 1 Department of Physiology and Biophysics, University of Arkansas for Medical Sciences, 4301 W. Markham Street, Little Rock, AR 72205, USA; 2 Department of Pathology, University of Arkansas for Medical Sciences, 4301 W. Markham Street, Little Rock, AR 72205, USA; 3 Arkansas Children's Nutrition Center, 15 Children's Way, Little Rock, AR 72205, USA; 4 USDA Agricultural Research Service, 15 Children's Way, Little Rock, AR 72205, USA; 5 Current address : Myeloma Institute for Research and Therapy, University of Arkansas for Medical Sciences, Little Rock, AR 72205, USA; 6 Current address : Center for Animal Nutrigenomics and Applied Animal Nutrition, Alltech Inc., Nicholasville, KY 40356, USA

## Abstract

**Background:**

Blueberries may lower relative risk for cancers of the gastrointestinal tract. Previous work indicated an inhibitory effect of consumed blueberry (BB) on formation of aberrant crypt foci (ACF) in colons of male Fisher F344 rats (inbred strain). However, effects of BB on colon tumors and in both genders are unknown.

**Methods:**

We examined efficacy of BB in inhibition of azoxymethane (AOM)-induced colon ACF and intestine tumors in male and female Sprague-Dawley rats (outbred strain). Pregnant rats were fed a diet with or without 10% BB powder; progeny were weaned to the same diet as their dam and received AOM as young adults.

**Results:**

Male and female rats on control diet had similar numbers of ACF at 6 weeks after AOM administration. BB increased (*P *< 0.05) ACF numbers within the distal colon of female but not male rats. There was a significant (*P *< 0.05) diet by gender interaction with respect to total colon ACF number. Colon and duodenum tumor incidences were less in females than males at 17 weeks after AOM. BB tended (0.1 > *P *> 0.05) to reduce overall gastrointestinal tract tumor incidence in males, however, tumor incidence in females was unaffected (*P *> 0.1) by BB. There was a tendency (0.1 > *P *> 0.05) for fewer adenocarcinomas (relative to total of adenomatous polyps plus adenocarcinomas) in colons of female than male tumor-bearing rats; in small intestine, this gender difference was significant (*P *< 0.05). BB favored (*P *< 0.05) fewer adenocarcinomas and more adenomatous polyps (as a proportion of total tumor number) in female rat small intestine.

**Conclusion:**

Results did not indicate robust cancer-preventive effects of BB. Blueberry influenced ACF occurrence in distal colon and tumor progression in duodenum, in gender-specific fashion. Data indicate the potential for slowing tumor progression (adenomatous polyp to adenocarcinoma) by BB.

## Background

Epidemiological findings generally indicate a lowering of relative risk of colorectal cancers in humans who regularly consume large amounts of fruits [1-7]. Conversely, individuals consuming relatively small amounts of fruits may have increased risk of colon cancer [8]. Fruit consumption lowers relative risk for cancers of the colon [7,9,10], rectum [11], and small intestine [12]. Protective effects of fruits may be more robust for cancers of the colon than rectum [2,10,13], may be stronger in distal than proximal colon sub-sites [2,13,14], and in at least one study were more prominent in men than women [10]. Adenomatous polyps (adenomas), the precursors to colorectal adenocarcinomas, are less prevalent in high fruit consumers [13,15]. In addition, colorectal adenoma growth was inversely associated with consumption of fruits and berries [16]. In spite of the above, there are few epidemiological studies that have examined for effects of specific types of fruits and in particular berries on human colorectal cancer risk.

Berry fruits contain a diverse array of phytochemicals that may have human health benefits [17]. The possible colon cancer-preventive actions of berries have received recent interest [18]. Extracts of berries inhibited, in dose-dependent fashion, proliferation of human cancer cell lines [19]. Among the phenolic compounds of the blueberry (BB), the anthocyanins were shown to be the most potent inhibitors of cell proliferation and inducers of apoptosis when tested, in relatively pure form, on colon cancer cell lines [20,21]. In humans consuming blueberries, a significant portion of the ingested anthocyanins reach the colon [22] where they may contribute to tissue anti-oxidant capacity and hence, to anti-carcinogenesis. Additionally, consumption of BB results in postprandial increases in plasma antioxidant level in humans [23]. Pterostilbene, another phytochemical constituent of blueberry, impedes advancement of the cell cycle and induces apoptosis of human cancer cell lines *in vitro *[24].

*In vivo *studies of the colon cancer-preventive actions of berries have used the AOM-administered male Fisher 344 rat or the Apc^min ^mutant mouse. Freeze-dried black raspberries in the diet inhibited the size (and therefore development) of colon aberrant crypt foci (ACF) as well as tumor multiplicity in male Fisher 344 rats [25]. In this same animal model, blueberry suppressed the total number of colon ACF per rat [26], similar to that observed with 40 ppm of pterostilbene added to diet [27]. In the Apc^min ^mouse model, the blackberry anthocyanin, cyanidin-3-glucoside, as well as an anthocyanin enriched extract of bilberry decreased numbers of small intestine adenomas [28]. Also in Apc^min ^mice, supplementation of diet with bilberry, lingonberry or cloudberry led to reductions in overall number and size of intestinal adenomas [29]. Feeding tart cherries or anthocyanin extract of cherries to Apc^min ^mice inhibited the frequency and size of intestine adenomas [30,31]. Thus, suppressive effects of berries on small intestine adenomas in Apc^min ^mice appear to be robust and consistent between studies.

Despite the above support for preventive effects of fruits and berries on colorectal cancers, several caveats remain. The *in vitro *studies typically observed effects of anthocyanins in the micromolar range, concentrations that may never be reached systemically or within the lumens of the small intestine or colon. The Apc^min ^mouse does not model sporadic colon cancers, the most frequent type of colorectal cancer. The AOM-treated, inbred Fisher 344 male rat is highly sensitive to dietary and chemical manipulation of colon carcinogenesis and many structurally diverse molecules have been shown to inhibit or promote ACF and/or tumors in this model [32]. Therefore, results from the above animal models should be examined in outbred rat strains and in both genders.

Insulin and insulin resistance have been positively linked with AOM-induced carcinogenesis in rats [33,34]. Serum C-peptide, a bio-marker of insulin secretion, is positively associated with colorectal cancer risk in humans [35] and is regulated by dietary factors [36]; however, relevance of insulin to suppressive effects of fruit consumption on colorectal cancer development is unclear. In this study, we used a validated, AOM-treated Sprague-Dawley (outbred) rat model (males and females) to evaluate effects of BB on intestine tumor genesis and serum C-peptide. Results did not provide strong support for cancer-preventive effects of BB in the rat colon.

## Methods

### Materials

Freeze-dried, powdered blueberry ['HiActives' Wild Blueberry Powder, Lot #01351573, derived from wild blueberries (*Vaccinium angustifolium*)] was obtained from FutureCeuticals Inc. (Momence, IL). Composition of blueberry (BB) powder was 2.73% protein, 5.5% fiber, 3.08% fat and 2.96% moisture. BB contained over 20 individual anthocyanins; the total amount of anthocyanins was ~28 mg/g dry weight. AIN-93G diets were formulated as in Table 1. The 10% BB dose was chosen in order to maximize consumption of anthocyanins and without significantly affecting food consumption when compared to the control diet.

**Table 1:** Experimental AIN-93G diets

Component (g/kg)		Control	Blueberry (BB)
Casein		200	196.3
L-Cystine		3	3
Corn Starch		397.5	310.3
Maltodextrin		132	132
Sucrose		100	100
Corn Oil		70	69
Cellulose		50	41.9
AIN93G Mineral Mix		35	35
AIN93G Vitamin Mix		10	10
Choline Bitartrate		2.5	2.5
TBHQ		0.014	0.014
Blueberry Powder		0	100
Total		1000	1000

### Animals

**Figure 1:**
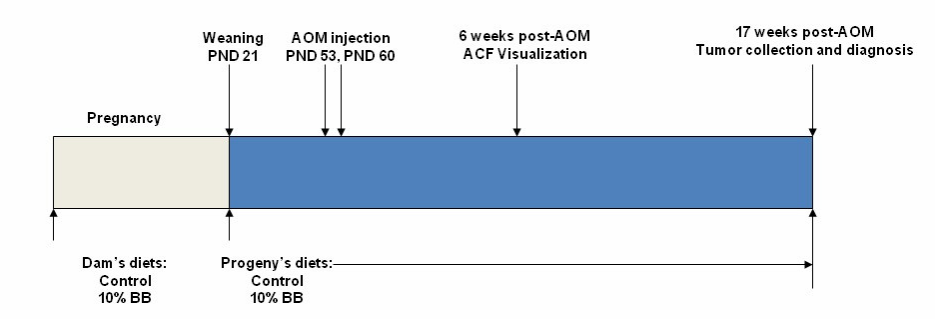
**Experimental design**. Gastrointestinal tissues were collected at 6 and 17 weeks after the second AOM administration for determination of ACF and tumors, respectively. PND, postnatal day.

Animal use protocols were approved by the University of Arkansas for Medical Sciences Institutional Animal Care and Use Committee. The experiment was designed to examine effects of "lifetime" BB diet exposure (including prenatal and pre- and post-weaning periods) on azoxymethane (AOM)-induced carcinogenesis. This experimental design is presented in Figure 1. Forty-four pregnant (gestation day 4) Sprague-Dawley rat dams (Charles River Laboratories; Wilmington, MA) were received in the Association for Assessment and Accreditation of Laboratory Animal Care - approved animal facility at the Arkansas Children's Hospital Research Institute. Dams were immediately placed on diets [Control (n = 22) or 10% BB (n = 22)]. At postnatal day (PND) 3, pups were weighed and litters culled to five males and five females. Pups were weaned at PND 21 and given the same diet as their dam for the duration of the experiment. After lactation, dams were used for studies not described here. Some offspring (randomly selected) were used for studies of adiposity and metabolism that also were outside the purview of the present focus and hence are not described here. Animals were housed in polycarbonate cages and permitted *ad libitum *access to food and water. Animal rooms were temperature- and humidity-controlled with a 12-h light-dark cycle.

### Tumors and ACF

AOM (Midwest Research Institute; Kansas City, MO) was administered subcutaneously (15 mg/kg body weight) to offspring at PND 53 and again at PND 60. Animals were weighed weekly throughout the duration of the experiment. ACF are observed in the colon mucosa during AOM-induced colon tumorigenesis and are used as an intermediate end point to evaluate cancer-prevention by nutritional and chemo-preventive factors [32]. Colons were obtained from 15 randomly selected animals of each diet group and gender at 6 weeks after the second AOM administration and used for visualization of ACF. Each colon was divided into three equal-length segments, designated as proximal, mid or distal. Segments were stained with 0.2% methylene blue in PBS for 3-5 minutes, rinsed with phosphate-buffered saline (PBS) for 1-2 minutes, and placed in 0.4% formalin-PBS. ACF were visualized using a Nikon AMZ800 stereoscope (40 × magnification) by a single observer blinded to treatment. ACF were elevated (single or multiple clustered) crypts with elongated and enlarged openings. The regional assignment of each ACF and number of crypts per ACF were recorded [37].

At 16 weeks post-AOM, a group of 10 animals from each diet group and gender were randomly chosen and subjected to necropsy for confirmation of tumor presence. At 17 weeks post-AOM, the liver, stomach, spleen, small intestine, cecum, colon and rectum from 73 or 74 animals of each diet group and gender were obtained and inspected for presence of tumors. Tumor location was determined relative to the distal end of the colon or small intestine. Tumors were removed, weighed, marked on the serosal surface with India ink, and placed in formalin. Serum was prepared from each animal. Pancreas and liver weights were recorded.

Small intestine and colon tumors were classified as adenomatous polyp (adenomatous polyp or adenomatous polyp with carcinoma *in situ*) or adenocarcinoma (invasive adenocarcinoma, invasive mucinous carcinoma, or metastatic adenocarcinoma with signet ring features) as described previously [36]. Diagnostic features of adenomatous polyps were well differentiated epithelial cells with low mitotic rate and absence of invasion. Adenocarcinomas were poorly differentiated, with cellular and nuclear pleomorphism, high mitotic rate, and evidence of invasion. Some colons manifested nodules which had a tumor-like appearance on gross examination, but which were comprised of focal areas/aggregates of lymphoid B and T cells [38]. These lesions, as well as the few observed mammary, liver, spleen, stomach, cecal and rectal tumors were excluded from statistical analysis.

### C-peptide

Serum C-peptide levels were measured using a rat C-peptide radioimmunoassay (Linco Research, Inc, St. Charles, MO). Sera (100 μl) from n = 12 to 14 animals of each diet group and each gender at 17 weeks post-AOM were assayed in duplicate in two replicate assays. Sensitivity of the assay was 25 pM and the inter-assay variation was less than 10%.

### Statistics

Statistical analysis was performed using SigmaStat for Windows, version 3.11 (Systat Software, Inc., Richmond, CA). BB effects on AOM-induced tumor incidence at 17 weeks post-AOM were examined by *χ*^2 ^analysis. Tumor pathology was compared for effects of diet and gender using Fisher's Exact Test. The effects of BB on colon ACF number and ACF crypt multiplicity (number of crypts per ACF) were evaluated by Student's *t *test or the Mann-Whitney rank sum test, the latter when data were not normally distributed. The interaction between effects of diet and gender on ACF was determined using two-way ANOVA. Effects of BB on serum C-peptide concentration were evaluated using Student's *t *test. All values are presented as means ± S.E.M. Differences between means were considered to be significant at *P *< 0.05, whereas 0.1 > *P *> 0.05 was considered to represent a tendency for a difference.

## Results

**Figure 2:**
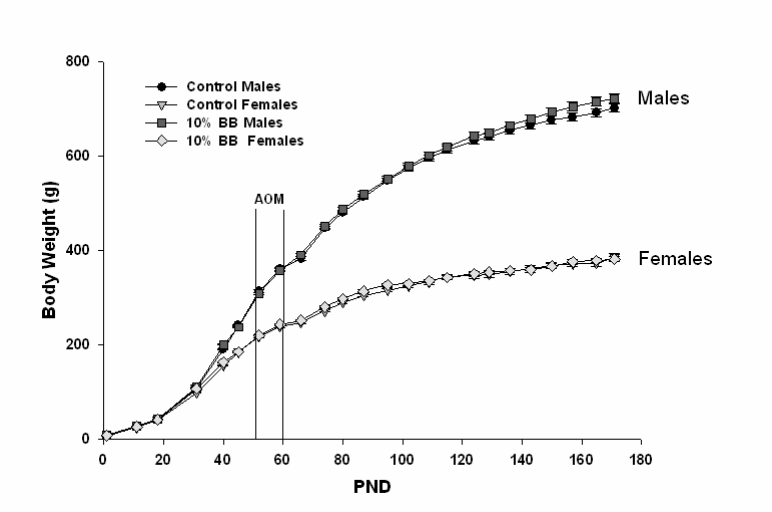
**Postnatal growth of experimental animals**. Body weights (mean and SEM) are shown. PND, postnatal day.

As expected, female rats gained less weight over time than did male rats (Figure 2); presumably due to gender differences in locomotor activity, blood androgen levels, insulin sensitivity and nutrient uptake/utilization, among other physiological differences. However, inclusion of BB to the diet had no effect on body weight accretion by rats of either gender (Figure 2); indicating no effect of BB on food consumption by either sex. BB had no effect on lengths of colon or rectum or weights of pancreas or liver (tissue weights were normalized to body weight) within each gender at 17 weeks post-AOM (data not shown). BB also had no effect on serum C-peptide concentration, an indicator of steady-state insulin secretion; however, C-peptide levels were ~30% lower for female compared to male rats (data not shown). Tumor status (presence or absence of tumors) had no effect on C-peptide concentration.

At 6 weeks after the second AOM treatment, male and female rats on the control diet had similar numbers of ACF (Tables 2, 3). ACF were most prevalent in mid, followed by distal colon; while proximal colons had fewer ACF. Addition of BB to the diet resulted in fewer ACF in mid and distal colons of male rats; however, results were not statistically significant (Table 2). Contrary to our hypothesis, BB consumption led to increased (*P *< 0.05) ACF numbers within the distal region of female rat colons (Table 2). ACF data were grouped by size, summed for each colon, and analyzed for effects of diet (Table 3). There was a tendency (0.1 > *P *> 0.05) for a ~24% reduction in total number of ACF in the colons of BB-fed male rats. In female rats, BB tended (0.1 > *P *> 0.05) to increase (by ~30%) the number of small ACF (Table 3). A significant (*P *< 0.05) gender by diet interaction was observed for occurrence of small and small plus large ACF (Table 3). BB and gender did not affect ACF crypt multiplicity (no. crypts per ACF) in male or female rat colons (Table 3).

**Table 2:** Colon ACF number (per rat) at 6 weeks after second carcinogen treatment.

	**Male**		**Female**	
	
Region	**Control**	**10% BB**	**Control**	**10% BB**
	
Proximal Colon	1.9 ± 0.6	2.2 ± 0.7	2.4 ± 0.8	2.7 ± 0.8
Middle Colon	92.7 ± 12.6	74.3 ± 9.0	77.7 ± 11.8	103.1 ± 9.9
Distal Colon	47.1 ± 5.6	34.7 ± 3.0	42.1 ± 5.4 ^a^	58.1 ± 5.3 ^b^

^a, b^indicate a significant difference between groups (*P *< 0.05); n = 15 animals of each gender for each diet group.

**Table 3:** Colon ACF number and multiplicity at 6 weeks post-AOM *

	Male	Female
**Number of Small ACF**		
Control	129.5 ± 15.4	117.6 ± 15.1 ^a^
10% BB	99.9 ± 8.9	152.8 ± 11.9 ^b^
Interaction between diet and gender	*P *= 0.016	

**Number of Large ACF**		
Control	12.2 ± 2.4	7.1 ± 2.5
10% BB	8.1 ± 1.7	10.1 ± 2.2
Interaction between diet and gender	NS	

**Total Number of ACF**		
Control	141.7 ± 16.7 ^a^	124.7 ± 15.2 ^a^
10% BB	107.9 ± 10.0 ^b^	162.8 ± 12.8 ^b^
Interaction between diet and gender	*P *= 0.012	

**ACF Crypt Multiplicity**		
Control	2.1 ± 0.1	1.9 ± 0.1
10% BB	2.0 ± 0.1	1.9 ± 0.1
Interaction between diet and gender	NS	

*Small ACF includes those with 1, 2 or 3 crypts; large ACF are those with 4 or more crypts; total ACF = no. small ACF + no. large ACF; ACF crypt multiplicity = no. of crypts per ACF.

^a, b ^within a column represents 0.1 > *P *> 0.05, student's t-test; also shown are *P *values for overall interaction between diet and gender as determined by two-way ANOVA.

n = 15 animals per gender for each diet group.

NS: *P *> 0.1

We evaluated tumor parameters at 17 weeks after AOM treatments, a time-point when tumors are prevalent in large and small intestines of this animal model (39). Colon tumor incidence was lower in female compared to male rats, regardless of diet (Table 4). Tumor incidence in small intestines (duodenum) also was reduced for female rats, regardless of diet. Comparing data summed for all segments of the colon and small intestine, BB tended (0.1 > *P *> 0.05) to reduce tumor incidence in males (Table 4). In contrast, tumor incidence in female rat colon and small intestine was unaffected (*P *> 0.1) by BB (Table 4).

**Table 4:** Incidence of AOM-induced tumors at 17 weeks post-AOM *

	Male		Female		
**# rats per group**	**Control** **(73)**	**10% BB** **(74)**	**Control** **(74)**	**10% BB** **(74)**	

					**Gender effect**

Proximal colon	5.5 (4)	4.1 (3)	1.4 (1)	2.7 (2)	NS
Middle colon	31.5 (23)	20.3 (15)	8.1 (6)	10.8 (8)	*P *< 0.001
Distal colon	10.9 (8)	10.8 (8)	0 (0)	2.7 (2)	*P *< 0.01
**Entire colon**	41.1 (30)	29.7 (22)	9.5 (7)	16.2 (12)	*P *< 0.001

Proximal SI **	23.3 (17)	12.2 (9)	6.8 (5)	6.8 (5)	*P *< 0.01
Middle SI	1.4 (1)	1.4 (1)	0 (0)	0 (0)	NS
Distal SI	0 (0)	0 (0)	0 (0)	0 (0)	NS
**Entire SI**	24.7 (18)	13.5 (10)	6.8 (5)	6.8 (5)	*P *< 0.01

**Colon + SI**	56.2 (41) ^a^	41.8 (31) ^b^	16.2 (12)	23.0 (17)	*P *< 0.001

* Incidence is percentage of tumor-bearing animals; actual number of animals with each tumor is shown in parentheses; gender effects were calculated from combined diet groups; six rectal tumors, two mammary tubular adenomas, one biliary cystadenoma, two cecal tumors, one stomach tumor, one spleen tumor, and a number of nodules representing focal areas of lymphoid hyperplasia [38] also were found; all were excluded from calculations of small intestine and colon tumor indices.

** SI: small intestine (almost all tumors were found in duodenum; with the exception of two in the jejunum).

^a, b ^indicate a tendency for a difference (*P *= 0.081); NS: *P *> 0.1

**Figure 3:**
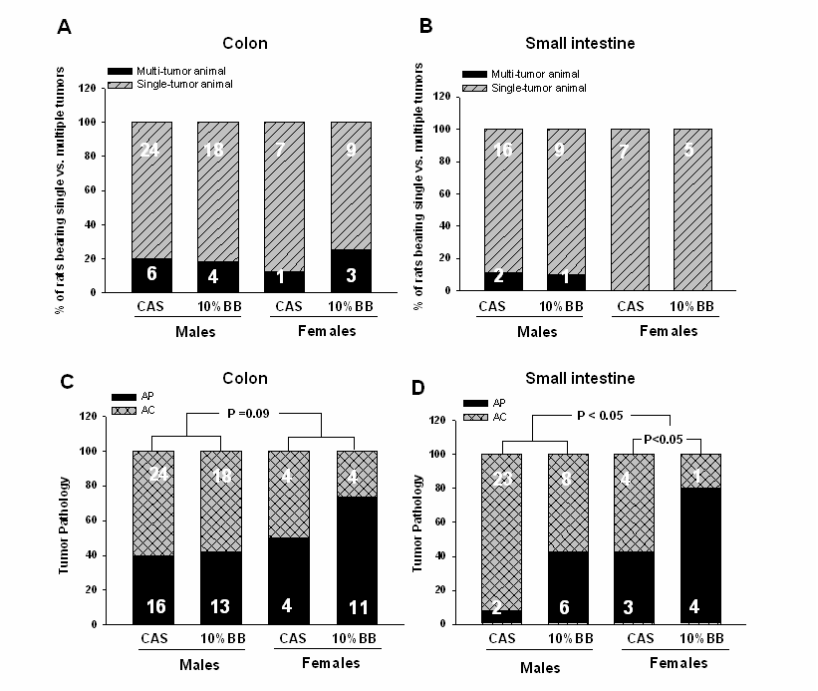
**Tumor multiplicity and tumor pathology for experimental animals**. The relative proportion of tumor-bearing animals having single or multiple (> 1) tumors in the colon (*A*) or small intestine (*B*) was determined. Relative proportion of tumors classified as adenomatous polyps (AP) or adenocarcinomas (AC, this group also included AC with signet ring features) in colon (*C*) and small intestine (*D*) is presented as percentage of AP + AC. n = 73 (control male rats) or n = 74 (10% BB male and female rats, control female rats). Actual numbers of animals having one or greater than one tumor also is indicated in *A *and *B*. Actual numbers of AP and AC tumors also are indicated in *C *and *D*.

BB had no effect on tumor multiplicity (# tumors per tumor-bearing rat) in colon or small intestine and this measure was also unaffected by gender (Figure 3A, B). Similarly, no differences in tumor weight were apparent between diets or gender (data not shown). The ratio of number of adenocarcinomas to the total number of adenomatous polyps plus adenocarcinomas was determined as an index of tumor progression. In the colon, there was a tendency for fewer adenocarcinomas in female than male rats; in small intestine, the gender difference was significant (*P *< 0.05) (Figure 3C, D). BB diet favored fewer adenocarcinomas and more adenomas (as a proportion of total tumor numbers) in female rat small intestine (*P *< 0.05).

## Discussion

Our results did not provide strong support for a beneficial role of BB in inhibition of AOM-induced intestinal cancers in Sprague-Dawley rats. BB only tended to reduce the total number of colon ACF and overall intestine tumor incidence and only in males. In female rats, BB augmented ACF numbers in distal colon, contrary to the hypothesized protective action. The non-significant tendency for a reduced number of ACF in male Sprague-Dawley rats consuming BB diet somewhat agrees with findings by Boateng *et al.*, 2007 [26], who studied male Fisher 344 rats. However, the latter group reported a statistically significant and robust suppression of ACF by BB diet, in contrast to that observed here. This difference between studies may reflect the two rat strains used, the differences in group size (n = 6 animals for previous study, n = 15 animals for present study), and/or the differing amounts of blueberry incorporated in the diet (10% in the present study, 5% in the previous study). With regard to differences in dietary content of blueberry, we do not consider this a likely cause of the study differences, since anthocyanins suppressed adenoma formation in a linear dose-dependent fashion in Apc^min ^mice [28]. It is plausible that Sprague-Dawley rats are less susceptible (and/or individually more variable) to dietary influences on AOM-induced ACF and tumor formation than inbred Fisher 344 rats, which also may contribute to differences between studies. In marked contrast to these findings was the significant increase in ACF occurrence in distal colon of female rats consuming BB. The basis for this novel, gender-specific response to BB is unknown, albeit, we speculate that this may involve functional interactions of BB constituent(s) with female sex hormones and/or receptors.

Regional distribution of adenocarcinomas closely followed that for ACF, as in our prior studies [36,37,39]. Specifically, ACF and tumors were more prevalent in mid > distal > proximal colon. Several epidemiological studies reported more pronounced cancer-protective effects of fruits in distal rather than proximal colon of humans [2,13,14]. The functional correspondence of the mid colon of the rat to one or more sub-sites of the human colon is not known; anatomically, it corresponds to transverse and upper distal regions of the human colon. The lack of inhibitory effect of BB on tumorigenesis in the rat colon may suggest that BB will not be preventive for human colon cancers, although epidemiological studies are necessary to resolve this question.

The similar ACF numbers for male and female rats on control diet, but less frequently occurring colon tumors in females relative to males, were noted. Thus, progression of ACF to adenomatous polyps may be slowed or partially blocked in female rats or alternatively, ACF in female rats may regress to a greater extent during the later stages of tumorigenesis, also leading to fewer polyps. Such mechanisms have a precedent in the case of AOM-treated AKR/J mice which are resistant to colon tumor-formation but manifest ACF in their colons [40]. Striking differences in tumor incidence with gender were maintained across the three regions of the colon as well as proximal small intestine (duodenum). This is consistent with previous observations of greater tumor incidence in male than female rats after administration of colon carcinogens [41-43]. Since ACF numbers were similar for males and female Sprague-Dawley rats on control diet, liver activation of AOM to the proximate carcinogen, methylazoxymethanol [44] likely does not differ with gender. Rather, the involvement of systemic or local factor(s) that affect post-initiation phases of intestinal tumorigenesis and which exhibit sexual dimorphism is suggested. We speculate that insulin may be one such factor, since C-peptide levels were significantly lower in female rats (presumably as a consequence of their increased insulin sensitivity) and insulin (and insulin resistance) have been positively linked with AOM carcinogenesis in rats [33,34]. Interestingly, incidence of colorectal cancer is slightly lower in women than men [45]. Thus, a further understanding of how gender affects tumorigenesis in this animal model may be relevant to elucidating endocrine and other influences on human colorectal cancer incidence rates.

Regional differences in tumor incidence within the colon and small intestine have been reported [36,37,39,46,47] and were confirmed here. Another laboratory has shown in male Sprague-Dawley rats that AOM induces DNA modifications (alkylation) to similar extents in proximal and distal halves of the colon; however, the distal half of the colon responds to these DNA modifications with a greater degree of apoptosis and DNA repair, compared to proximal colon [46]. In view of the known apoptosis-inducing effects of anthocyanins and stilbenes *in vitro*, we hypothesized that these molecules, when provided via BB diet, would promote apoptosis of colon epithelial cells whose DNA was newly modified by AOM during initiation of carcinogenesis, thus resulting in fewer ACF and tumors. However, our ACF and tumor results did not support this hypothesized action. Perhaps, the levels of these bio-active factors within the microenvironment of the colonic crypt and luminal epithelium were limiting.

Consumption of fruits may protect against cancer development in human small intestine [12]. Indeed, we observed that BB inhibited the adenoma-adenocarcinoma transition in female rat duodenum. This is generally concordant with the reported inhibition, by fruit consumption, of colorectal adenoma growth in humans [13,15,16] and the rather consistent inhibition by berries of adenoma growth in Apc^min ^mice [29-31]. In the latter model, adenomas are common in small intestine. Thus, collective findings highlight the potential for use of berries and fruits in dietary prevention of small intestine tumors.

*In vitro *and *in vivo *studies implicate anthocyanins and other phytochemicals in growth-inhibition of cancerous cells [20,21,24,27]. Such molecules may have estrogenic or anti-estrogenic actions [48] which may pertain to the gender-specificity of effects observed, although this is speculative. Further studies that examine how BB components affect the adenoma-adenocarcinoma transition, and how this is affected by gender, may yet reveal approaches for cancer-prevention using whole BB or its constituent(s), alone or in combination with other colon cancer-preventive foods or factors.

## Conclusion

Results did not demonstrate robust cancer-preventive effects of BB but identified marked gender differences in incidence of AOM-induced tumors. Blueberry influenced, in gender-specific fashion, ACF occurrence in distal colon and tumor progression in duodenum. These findings illustrate the complex interplay of bio-active dietary factors, gender, tissue sub-site, and endocrine status in intestinal carcinogenesis and neoplasia.

## Abbreviations

BB: blueberry; AOM: azoxymethane; ACF: aberrant crypt foci; PBS: phosphate-buffered saline.

## Competing interests

The authors declare that they have no competing interests.

## Authors' contributions

FAS and RLP conceived and planned the study. FAS drafted the manuscript. JAF quantified ACF, performed C-peptide assays and analyzed the data. XW helped with experimental planning and diet formulation. RX participated in tumor collection and performed data analysis. LJH conducted all pathology. All authors provided input during manuscript revision and all authors read and approved the final manuscript.

## Pre-publication history

The pre-publication history for this paper can be accessed here:

http://www.biomedcentral.com/1471-230X/9/67/prepub
